# Development and psychometric testing of the nutritional and social health habits scale (NutSo-HH): A methodological review of existing tools

**DOI:** 10.1016/j.mex.2024.102768

**Published:** 2024-05-22

**Authors:** Elena Sandri, Michela Piredda, Maddalena De Maria, Stefano Mancin, Marco Sguanci, Asensi Cabo, Germán Cerdá Olmedo

**Affiliations:** a Faculty of Medicine and Health Sciences, Catholic University of Valencia San Vicente Mártir, c/Quevedo, 2, Valencia 46001, Spain; b Doctoral School, Catholic University of Valencia San Vicente Mártir, c/Quevedo, 2, Valencia 46001, Spain; c Research Unit Nursing Science, Campus Bio-Medico di Roma University, Via Alvaro del Portillo 21, 00128 Rome, Italy; d Department of Life Health Sciences and Health Professions, Link Campus University, Via del Casale di San Pio V, 44, 00165 Rome, Italy; e IRCCS Humanitas Research Hospital, Via Manzoni 56, 20089 Rozzano – Milan, Italy; f Clinical Psychologist, Onda Town Council, Career Civil Servant, c/El Pla 1, Onda-Castellón, 12200, Spain

**Keywords:** Health determinants, Methodological review, Nutrition, Scale validation, Spanish Population

## Abstract

Habits represent repeated patterns of behavior over time that exert a significant influence on individual health. While specific tools exist to measure individual habits, the number of instruments capable of simultaneously exploring multiple dimensions of health is limited. This research had two main objectives: 1) to examine the literature to find existing tools for evaluating health habits, especially in the Spanish population; 2) through a methodological review, to develop and validate a tool capable of measuring multiple dimensions of health habits. The Nutritional and Social Health Habits Scale (NutSo-HH) was conceived, tested, and refined through pilot testing with cognitive interviews and expert content validation. Construct validity was explored through confirmatory factor analysis and known-group validity, while criterion validity was verified in comparison with the ``Healthy Nutrition Index for the Spanish Population.'' Reliability was assessed using omega coefficients. Confirmatory factor analysis yielded satisfactory fit indices. The final model included two second-order factors (nutritional habits and health habits) and two first-order factors (Mediterranean diet and alcohol consumption). Omega coefficients ranged from 0.521 to 0.815. The NutSo-HH Scale emerges as a valid and reliable tool to assess nutritional and social habits among Spanish young adults. This novel instrument fills a gap in the field, allowing exploration of various health determinants through a single scale and providing support for decision-making in the realm of public health nutrition.

## Review Highlights

• Existing literature provides specific scales for individual habits, highlighting a lack of comprehensive and multidimensional tools for a holistic assessment of various health dimensions within a population.
• Through methodological review and exploration of existing literature, NutSo-HH was developed and validated as a tool capable of measuring multiple dimensions of health habits.
• This novel instrument allowed the exploration of various health determinants through a single scale, providing support for decision-making in the fields of nutrition and public health.

## Graphical abstract

**Figure:**
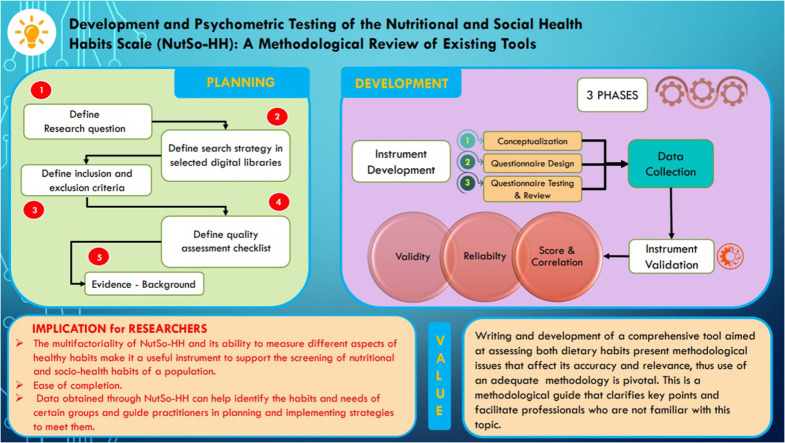

Specifications table

**Table:** 

Subject area:	Medicine and Dentistry
More specific subject area:	Public Health, Nutritional and Lifestyle determinants
Name of the reviewed methodology:	Methodological review of existing instruments and health determinants scales and development of a new comprehensive instrument.
Keywords:	Health Determinants, Methodological Review, Nutrition, Spanish Population, Scale Validation
Resource availability:	A. Bach, L. Serra-Majem, J.L. Carrasco, et al. The use of indexes evaluating the adherence to the Mediterranean diet in epidemiological studies: a review, Public Health Nutr 9 (2006) 132–146. https://doi.org/10.1079/PHN2005936.
Review question:	• To what extent do existing tools in the literature allow for a comprehensive and multidimensional study of the health status within a population? • Is the creation of a comprehensive tool capable of effectively describing the health status of a population feasible?

**Method details**

## Background

Youth and young adulthood is a sensitive and important period when major changes take place in individuals, and unhealthy nutritional behaviours and lifelong habits can also develop [1,2]. Most people in this age experience rapid weight gain [3] with its adverse consequences, such as cardiovascular diseases [4], some cancers [5] and obesity [6]. Several indices of diet quality related to food groups [7,8] or of the degree of adherence to a given dietary pattern [9,10] have been developed over the years. For instance, the Mediterranean Score (MS) [11], based on indicators on the consumption of certain food groups, provides a score of healthiness of the diet followed. The Diet Quality Index (DQI) [12], analyses different macromolecules (fatty acids, saturates, cholesterol, complex carbohydrates), minerals (sodium, calcium), food groups (fruits and vegetables, proteins) and their presence in smaller or larger quantities in diet. The Mediterranean Diet Adherence Screener (MEDAS) [13] includes a series of dietary habits, assigning a score 1 when Mediterranean diet is followed and 0 when it is not, with a minimum score needed to affirm that the subject adheres to the Mediterranean diet. However, the above indices quantify only nutritional aspects, while a dietary analysis alone does not provide a comprehensive view of the factors that affect the population health and well-being. While, it would be not feasible to use an instrument that encompasses all dimensions of such a multifaceted phenomenon as health, it would be useful to analyse as a whole diet and a series of healthy or unhealthy habits (such as sleep/rest, social habits, eating disorders) related to nutrition, whose practice can influence quality of life and the development of certain diseases in the population [14,15]. Sleep quality and duration are important for subjective well-being [16] and are related to adverse cardiometabolic risk [17], to poor academic performance [18] and affect people's emotions and feelings [19]. Several studies describe how the type of diet and nutrient intake affects sleep variables [20].

Eating disorders have recently become a major public health concern [21]. The contemporary social and cultural emphasis on thinness for women and muscularity for men, creates unrealistic stereotypes of what constitutes beauty, with pervasive media images of idealized bodies that lead to the global rise of eating disorders [22]. Although some instruments are available to detect the presence of eating disorders [21,23,24] such phenomenon affects health so profoundly that it must be analysed in a broader context, related to the adoption of other social and nutritional habits.

Evidence exists on the effects of alcohol consumption, particularly among young people going out at night who look to feelings of well-being and stimulation produced by alcohol [25]. Excessive alcohol consumption impairs psychomotor performance, disrupts cardiovascular function and sleep, and can alter mood and behaviour the next day [26]. Because alcohol is associated with numerous health problems [27,28], it is advisable to limit its consumption in the diet.

## Rationale and methodological review objective

The conception of a comprehensive tool aimed at assessing both dietary habits and those closely linked to health, such as eating disorders, sleep behaviors, leisure activities, and alcohol consumption, holds significant importance for analyzing the health status of a population. Consequently, following an in-depth literature review on existing tools for evaluating health habits, particularly in the Spanish population, a methodological review was conducted with the primary objective of developing and validating the NutSo-HH tool.

## Methods

The literature review has unveiled several questionnaires currently available for assessing dietary habits within a population [[7], [8], [9],[11], [12], [13]]. However, no instruments have been identified that enable a comprehensive collection of information across the broad spectrum of habits influencing health. To address this gap, a thorough methodological review of existing instruments was conducted with the aim of developing a novel and specific tool. A cross-sectional validation study was conducted in three phases. In the initial phase, the instrument was developed, and in the second phase it was administered to a sample of Spanish young adults residing in Spain. In the third phase, the recommendations of the European Statistical System for the development and validation of instruments [29] were followed.

### Phase 1: instrument development

#### Conceptualization

The definition of eating and social habits on which this study is based is the set of daily behavioural patterns of a person who have a certain repetition over time, and which influence the health and well-being of the person [30]. The theoretical framework used for the development of the dimensions of the instrument was based on several studies on eating habits in Spain [31,32] on the role of rest in health [16,33], on the effect of alcohol consumption [34,35] and on the incidence of eating disorders [36,37].

#### Questionnaire design

A literature search was conducted in PubMed and Google Scholar on 15 January 2020 to find existing instruments in the field of nutrition and health habits. The following key words: eating, nutrition, feeding behavior, food intake, diet, lifestyle, nutrition survey, surveys and questionnaires, were searched. They were used both as Mesh terms and text words, and opportunely combined through Boolean operators. The full search strategy used in PubMed is presented in Table S1, Supplementary file 1. Several instruments were found: Mediterranean food pattern PREDIMED Study (MeDiet-PREDIMED) [38], Cardioprotective Mediterranean diet index (Cardio) [39], Food-Mood Questionnaire (FMQ) [40], Global physical activity questionnaire (GPAQ) [41].

Based on the above, a pool of 27 questions was generated aimed at investigating frequency of consumption of different foods, rest habits, alcohol use and social habits. Specifically, nine items, inspired by the MeDiet-PREDIMED questionnaire [38], explored the consumption of foods characteristic of the Mediterranean diet. Three newly created, items asked about beverage consumption and other three explored the consumption of unhealthy foods. Still in the field of nutrition, three questions explored possible symptoms of eating disorders. In the field of lifestyle habits, three questions taken from the GPAQ questionnaire [41], explored the dimension of sedentary lifestyle and physical activity, three questions explored the habits of alcohol and tobacco consumption or partying at night and three questions focused on sleep and rest. Finally, to collect socio-demographic data, 13 questions were added. This phase of the research was conducted by three members of the research team with expertise in nutrition and health.

### Questionnaire testing and review

#### Cognitive interviews

To assess face validity and readability, the 27-item questionnaire draft was administered to a pilot sample (*n* = 52) of the target population. Structured cognitive interviews were conducted with six questions and two statements on completion time, clarity, readability, completeness, acceptability, and formal aspects of the questionnaire (Table S2). For questions 1–6 each respondent assigned a score from 0 to 5, where 0 was ‘not very suitable’ (not complete, not clear, etc.) and 5 was ‘very suitable’ (very complete, very clear, etc.). Regarding feedback statement 7, 96 % participants responded that they did not have any difficulties (*n* = 43) or did not answer (*n* = 7), and only 4 % (*n* = 2) provided the same criticism regarding two items referring to sport, which were then removed. As regards to feedback statement 8, 19 participants (37 %) made suggestions that were discussed during the group.

#### Content validity

Seven experts in nutrition and health promotion (two psychologists, a nutritionist, a social educator, two family doctors and a communication professional) assessed content validity of the instrument draft. First, the experts independently analysed the items and the results of the pilot survey and filled in a structured questionnaire with the same questions asked to the pilot group, together with questions 9, 10 and 11 of Table S2 (Supplementary file 1).

**Fig. 1:**
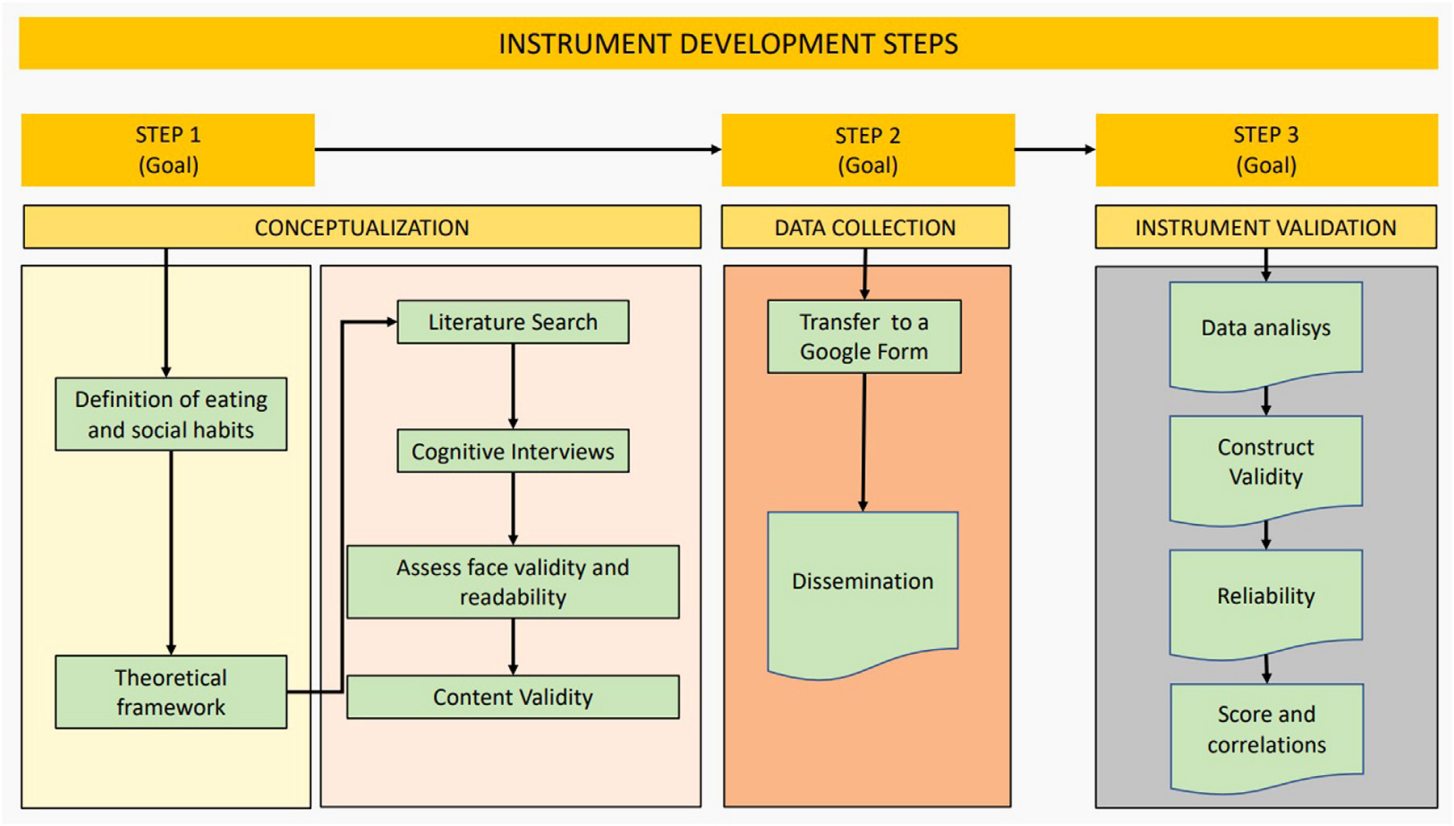
Flowchart of Instrument Development.

The expert suggested changes for 14 items and the introduction of 4 new items. Then the expert group met and discussed each item with related suggestions. To determine the final items to be included in the questionnaire, a Content Validity Index was computed at item level (I-CVI) and for the overall instrument as mean of all I-CVI (Scale Content Validity Index, S-CVI) [42]. The experts rated each item on a 4-point scale (from 1 ``not relevant'' to 4 ``very relevant''). The I-CVI was calculated as the ratio between the number of experts rating the item as 3 or 4 and the total number of experts. To adjust for chance agreement among ratings a I-CVI. A validity index greater than 0.78 was considered excellent [42]. Six items did not reach the minimum I-CVI and were eliminated. The S-CVI was 0.91. The final instrument was called ‘Nutritional and Social Health Habits Scale' (NutSo-HH) and consisted of 23 items, posing a 6-factor model. (See the supplementary File for the final version of the NutSo-HH Scale). Fig. 1 summarizes the main development phases of the tool.

### Phase 2: data collection

NutSo-HH was transferred to a Google Form to be easily disseminated through social networks and self-completed by participants. Non-probabilistic snowball sampling was conducted. The survey was disseminated between August 2020 and October 2020 through: the researchers' personal social networks; e-mails to the members of the Valencian Youth Institute; an e-mail explaining the research and a poster with a QR code to be displayed, was sent to several establishments throughout Spain (such as pharmacies, clinics, tobacconists, etc.) selected because of their heterogeneous public; a specifically created Instagram account (@elretonutricional), from which various professionals and influencers were contacted and invited to help with the dissemination.

The inclusion criteria were people aged 18–45 years, of Spanish nationality and resident in Spain. Exclusion criteria were people who had a chronic disease that could affect their diet and people who at the time of the survey were in a situation that temporarily deregulated their usual diet: hospitalization, prison admission, etc.

The sample included 571 young adults, mostly female (*n* = 467, 81.8%), with a mean age of 30 years, and high education level as 64.3 % held a university degree. Sample demographic data are described in more detail in Table S3 (Supplementary file 1).

### Phase 3: instrument validation

#### Data analysis

Descriptive statistics were calculated for socio-demographic variables and factors (Table S4 – Supplementary file 1). Although a sample size of 200 respondents was considered adequate to perform a confirmatory factor analysis, a sample of at least 500 subjects was sought to allow testing known group validity [43]. According to classical test theory [44], we tested the dimensionality of NUTSO—HH with confirmatory factor analysis (CFA) positing six dimensions as the instrument was model-driven according to a literature review. In fact, this approach is recommended when the instrument is developed according to a theory-based model, as in our case. Thus, we performed CFA to confirm the dimensionality of the scale. Specifically, a CFA, that uses the correlation matrix of observed variables based on linear relationships between observed variables and latent factors, was carried out by posing a six-factor model [45].

Considering the ordinal level of the items included in the scale it was decided to use the robust Weighted Least Square (WLS-MV), which is highly recommended for applied researchers to analyze variables with less than five ordered categories [45]. The model fit was tested with a multifaceted approach [46,47], using the following indices: chi-square (x2) test, the Root Mean Square Error of Approximation (RMSEA; values ≤0.05 indicating a well-fitting model), the Comparative Fit Index, the Tucker and Lewis Index (CFI and TLI; values≥ 0.95 indicate a good fit), and the Standardised Root Mean Square Residual (SRMR; values ≤ 0.08 indicate a good fit). The chi‐square statistics are also reported and interpreted; however, due to their sensitivity to sample size, they were not used in interpreting model fit. The modification indices were inspected in case of a misfit.

Construct validity was further examined by posing the following hypotheses that 1) younger people, with higher education and income would show greater scores for nutritional and healthy habits [48]; 2) people with normal BMI compared with lower or higher BMI, would score higher in nutritional and healthy habits [49]. To identify such differences between mean scores, one-way analysis of variance with post-hoc Tukey's was conducted.

Criterion validity of the new instrument was analysed through comparison with ‘Healthy Nutrition Index for the Spanish population’ (IASE) [50]. Correlations between the mean scores of the scale’ factors were evaluated through Pearson's product-moment correlation coefficients. Values of 0.10–0.29 were considered as small, 0.30–0.49 as moderate, and 0.50 as strong [51]. The reliability of the NutSo-HH was assessed by evaluating the internal consistency of each factor using the omega coefficient [52]. Values 0.70 were considered as adequate [53]. Significance was set at *p* < 0.05. Statistical analyses were performed using SPSS 26.0 (IBM Corp. Armonk, NY, USA) for descriptive statistics, Mplus 8.8 [54] for factorial analyses and R for ordinal-omega reliability coefficients.

#### Construct validity

We specified a six-factor confirmatory model: F1 Mediterranean foods (measured by items Q1, Q2, Q3, Q4, Q5 and Q6), F2 Healthy and unhealthy foods (measured by items Q7, Q8, Q9 and Q10), F3 Meat and dairy products (measured by items Q11, Q12, Q13 and Q14), F4 Eating disorders (measured by items Q15, Q16, Q17), F5 Alcohol consumption (measured by items Q18, Q19 and Q20), and F6 Rest habits (measured by items Q21, Q22 and Q23). Following inspection of the modification indices, the cross-loading of items Q2 and Q3 in F1 and F2 were specified in the model that yielded the following fit-of-goodness: *χ*2(213, *N* = 571) = 484.750, *p* < 0.0001; RMSEA= 0.047 (IC 90 % 0.042–0.053) *p* = 0.785; CFI= 0.949; TLI= 0.940; SRMR= 0.064.

**Fig. 2:**
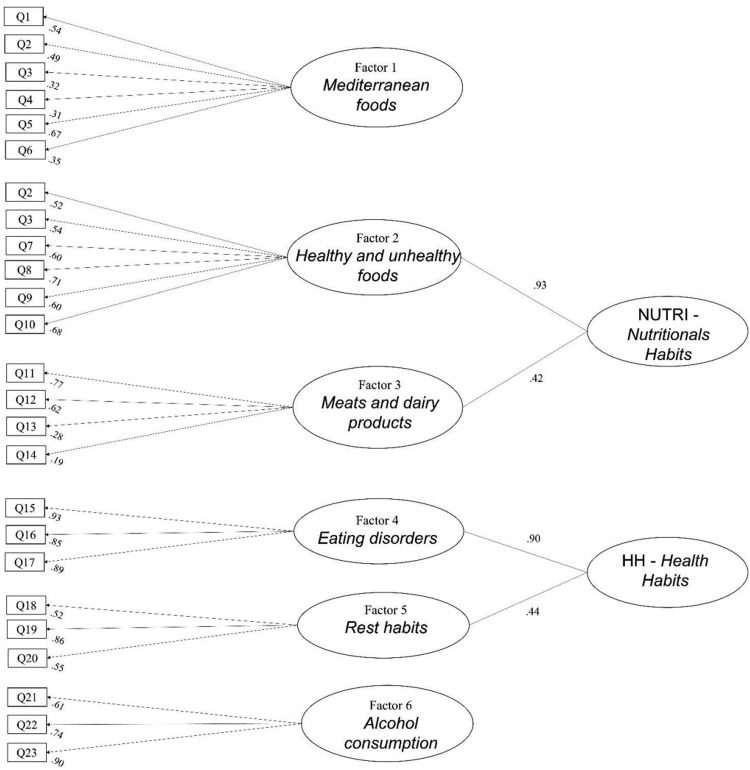
CFA and item loadings to factors.

Since the factors F2 - F3, and F4 - F5 were correlated (*r* = 0.368, *p* < 0.001 and *r* = 0.395, *p* < 0.001, respectively) we tested a more complex factorial structure composed of two second-order factors named Nutritional habits (NUTRI) and Health Habits (HH) and two first-order factors, F1 and F6. The Nutritional habits was loaded by Healthy and unhealthy foods and Meat and dairy products first-order factors. The Health Habits was loaded by Eating disorders and Alcohol consumption first-order factors. This model displayed the following fit indices: Chi-Square Test of Model Fit: (df: 218) = 501.388, *p* < 0.0001; RMSEA= 0.048 (IC 90 % 0.042–0.053) *p* = 0.747; CFI= 0.947; TLI= 0.939; SRMR= 0.068. Almost all the items showed loadings> 0.3 and significant p values< 0.001. Only items Q13 ‘White meat’ and Q14 ’Red meat’ showed loading 0.187 and 0.283, respectively (Fig. 2). The score of IASE correlated strongly with F1 Mediterranean foods (*r* = 0.728, *p* < 0.01), moderately with NUTRI (*r* = 0.446, *p* < 0.01), and weakly with HH (*r* = 0.174, *p* < 0.01) (Table S5 – Supplementary file 1).

#### Know-group validity

ANOVA showed significant differences in the mean scores of NUTRI (*p* = 0.001), HH (*p* < 0.001) and F1 (*p* = 0.012) according to BMI. In particular, better NUTRI, HH and F1 scores were shown in people with normal BMI compared to underweight or overweight people. Similarly, significant higher scores were found for NUTRI (*p* < 0.001), HH (*p* < 0.05) and F1 (*p* = 0.002) in people with at least university education compared with lower education, and higher scores of HH in people with higher compared with lower income, and higher scores of F1 in younger (18–30 years) compared with older (31–45) people (*p* = 0.036).

#### Reliability

To evaluate the internal consistency reliability, we computed the omega coefficient reliability for all the first-order factors and for the two second-order factors of the NutSo-HH on the total sample. The internal consistency reliability of the six factors representing diet and other habits related to nutrition and health was the following: Factor 1 = 0.6, 95 % CI 0.54–0.65; Factor 2 = 0.77, 95 % CI 0.74–0.80; Factor 3 = 0.57, 95 % CI 0.51–0.63; Factor 4 = 0.92, 95 % CI 0.91–0.93; Factor 5 = 0.70, 95 % CI 0.66–0.74; Factor 6 = 0.79, 95 % CI 0.77–0.82. The Omega reliability index was 0.70 (95 % CI 0.66–0.74) and 0.78 (95 % CI 0.75–0.81) for the Nutri and Health Habits factors. These results attested the internal consistency of all factors of the scale.

#### Scores and correlations

The mean scores of NUTRI, HH, F1 and F6 ranged 2.69–3.65 (1–4). NUTRI showed small significant correlations with HH, F1 and F6 (Supplementary file 1).

## Discussion and implication for researchers

The aim of this study was to review various nutritional scales present in the literature and, subsequently through a methodological review, to develop and psychometrically test a scale capable of measuring different aspects of health in a global way. The final instrument was called ``Nutritional and Social Health Habits Scale'' (NutSo-HH) (Supplementary file 2). The novelty and strength of this study is that it provides a new valid and reliable scale able to jointly measure two major aspects with an impact on health, namely nutrition and social habits. Based on this study results, the NutSo-HH can be used in the Spanish population for its purpose, as its validity and reliability were supported. Knowing the nutritional and social habits is essential for a better assessment of health and could allow clinicians to develop tailored intervention to enhance specific nutrition and health habits. Rigorous methodological approaches were followed for instrument development and psychometric testing. A hierarchical structure with two second order factors along to two first order factors was found. The complex factorial structure of the NutSo-HH was consistent with the literature [[55], [56]]. The NUTRI second-order factor comprises habits that regards two main aspects of nutrition habits, the consumption of Healthy and unhealthy foods and Meat and dairy products [[57], [58], [59], [60], [61]]. The HH second order-factor includes aspects such as rest habits and the presence of eating disorders, two very important aspects in the field of an individual's health [[62], [63], [64], [65], [66]]. Two items (Q2 ‘fruit‘ and Q3 ‘vegetables’), which in the original model were included in Mediterranean foods, loaded also in Healthy and unhealthy foods, meaning that their score contributes to the measure of both factors. This is not surprising, as fruit and vegetables are usually found both in the Mediterranean and in a generally healthy diet [67,68].

Further support to construct validity was provided by analysis of known-group validity. The theoretical hypotheses were confirmed, as scores of the new instruments differed according to respondent characteristics (BMI, age, education, income) in line with data from the literature [[69], [70], [71], [72], [73], [74]]. As expected, criterion-related validity with the IASE nutritional index was found for the ‘nutritional’ factors of NutSo HH, namely NUTRI (*r* = 0.446; *p* < 0.01) and F1 Mediterranean foods (*r* = 0.728; *p* < 0.01) In contrast, the factors HH and F6 investigating health habits were respectively slightly (*r* = 0.174; *p* < 0.01) or not correlated with IASE.

The internal consistency of the multidimensional scale measured through the omega reliability index, ranged 0.57 - 0.92, with Factor 1 and Factor 3 with indices below 0.7. The Omega coefficients are derived from the factor loadings and the residual variances estimated from the factor solution of the NutSo-HH. The estimate Omega coefficients presented low values for effect of four items (items Q3 and Q5, and Q14 and Q13 in factor 1 and 2, respectively) showing lower factor loadings and higher residual variances. However, we suggest further testing in a more diverse sample before we revise these items since they are extremely relevant for the construct measures. Such further testing could provide with more information on the value of these items in a broad population of people with diverse nutritional and social habits. Moreover, reliability coefficients of NutSo-HH are comparable to those of existing scales in this field [75,76]. Therefore, it is justified to calculate a score for each factor (NUTRI, HH, F1, F6) of NutSo-HH.

The factor NUTRI enables to calculate a nutrition healthiness score in terms of the frequency of consumption of various food groups, which have consequences for health. NUTRI includes, on one hand, the main sources of protein such as fish and white meat, whose abundant consumption (2–4 per week) is recommended [77,78], and that of plant foods such as fruit and vegetables, whose consumption is recommended on a daily basis. On the other hand, the consumption of fast, fried or ultra-processed foods and sugary soft drinks are also harmful to health [58,79].

The Factor HH allows to calculate a health score for some behavioural habits of the Spanish population. It includes sleep/rest, exploring the subject's duration and quality of sleep, and whether the subject wakes up rested. Sleep is an essential component of health, and its timing, duration, and quality are critical determinants of health. It is advisable to sleep 6.5 - 8 h a day. Lack of sleep can lead to a wide range of disorders, such as hypertension, obesity, type-2 diabetes, cardiovascular disease, and impaired immune functioning [80,81]. HH includes also eating disorders that should be detected early to enable timely and effective interventions [[82], [83], [84]].

The instrument created is powerful because data obtained through NutSo-HH can help identify the habits and needs of certain groups and guide practitioners in planning and implementing strategies to meet them. NutSo-HH could also be used to assess the impact of training and awareness-raising actions on nutritional and healthy habits carried out in a given target group. Future studies should provide further validation within more heterogeneous samples, including more male participants, with lower economic and educational status, and assessing stability over time and responsiveness.

## Limitations

Some limitations should be acknowledged. Firstly, despite the efforts to achieve a homogeneous sample, the respondents were mostly female, with income and education level higher than the average Spanish population. These sample characteristics may affect the nutrition and health-related behaviour reported and could limit the applicability of the scale. Secondly, the cognitive interviews were conducted during the COVID-19 pandemic when the state of confinement or by psychological factors such as fear, uncertainty or anxiety may have influenced some nutrition and health habits. Thirdly, instrument responsiveness and stability at repeated measures with test-retest were not investigated.

## Conclusions

In the literature, particularly in Spain, there was a lack of a scale that could measure different dimensions of population health in a unified way. The multifactoriality of NutSo-HH and its ability to measure different aspects of healthy habits make it a useful instrument to support the screening of nutritional and socio-health habits of a population. In addition, the ease of completion makes it suitable for wide dissemination through social networks. Health promotion professionals can use NutSo-HH to track the nutritional and health habits of young adults.

The development of a comprehensive tool aimed at assessing both dietary habits present methodological issues that affect its accuracy and relevance, thus using an adequate methodology is pivotal. This study provides a methodological guide that clarifies key points and facilitate professionals who are not familiar with this method.

## Ethics statements

The study was conducted in accordance with the Declaration of Helsinki and approved by the Ethics Research Committee of Catholic University of Valencia (approval code UCV/2019–2020/152). Informed consent was obtained from all subjects involved in the study.

## CRediT author statement

**Elena Sandri**: Conceptualization, Methodology, Data analysis, Writing – original draft, Writing – review & editing, Investigation, Validity tests. **Michela Piredda**: Methodology, Investigation, Data Analysis, Writing – original draft, Writing – review & editing, Validity tests. **Maddalena Di Maria**: Methodology, Validity tests, Writing – review & editing, Supervision. **Stefano Mancin**: Visualization, Writing – review & editing. **Marco Sguanci**: Visualization, Writing – review & editing**. Asensi Cabo**: Conceptualization, Methodology, Data Analysis, Writing – review & editing. **Germán Cerdá Olmedo**: Conceptualization, Methodology, Writing – review & editing, Supervision.

## Funding

This research did not receive any specific grant from funding agencies in the public, commercial, or not-for-profit sectors.

## Declaration of competing interest

The authors declare that they have no known competing financial interests or personal relationships that could have appeared to influence the work reported in this paper.

## Data Availability

Data will be made available on request.

## Appendix Supplementary materials

Image, application 1

Image, application 2
