# Supplementary material for: Development and psychometric testing of the nutritional and social health habits scale (NutSo-HH): A methodological review of existing tools
Source: MethodsX. 2024 May 22;12:102768. doi: 10.1016/j.mex.2024.102768 (PMC11177200; doi:10.1016/j.mex.2024.102768)
Supplement: Supplementary file 1 [file mmc1.doc]

**Supplementary File – Instrument Development Data**

Table S1: PubMed Search strategy.

| **PubMed Search strategy** |
| --- |
| ("diet, healthy"[MeSH Terms] OR "Nutrition Surveys"[MeSH Terms] OR "Eating"[MeSH Terms] OR "Feeding Behavior"[MeSH Terms] OR "Nutrition Surveys"[MeSH Terms] OR ("healthy diet*"[Title] OR "Healthy Eating"[Title] OR "Healthy Nutrition"[Title] OR "prudent diet*"[Title] OR "Healthy Eating Index"[Title] OR "Healthy Eating Indices"[Title]) OR ("Food Intake"[Title] OR "macronutrient intake*"[Title] OR "dietary intake*"[Title] OR "micronutrient intake*"[Title] OR "Ingestion"[Title] OR "feed intake*"[Title] OR "nutrient intake*"[Title] OR "nutritional intake*"[Title]) OR ("feeding behavior*"[Title] OR "eating behavior*"[Title] OR "feeding related behavior*"[Title] OR "feeding related behavior*"[Title] OR "dietary habit*"[Title] OR "diet habit*"[Title] OR "feeding pattern*"[Title] OR "food habit*"[Title] OR "eating habit*"[Title]) OR ("nutrition survey*"[Title] OR "nutritional survey*"[Title]) OR ("healthy life style*"[Title] OR "healthy lifestyle*"[Title])) AND ("Surveys and Questionnaires"[MeSH Terms] OR "questionnaires and survey*"[Title] OR "survey method*"[Title] OR "community survey*"[Title] OR "survey*"[Title] OR "instrument*"[Title] OR "tool*"[Title] OR "measure*"[Title] OR "scale*"[Title]) |

**Table S2**: Structured guide and scores for cognitive interviews and expert group.

| **Structured questions** | **Mean** | **Standard Deviation** |
| --- | --- | --- |
| **1. Is the time taken to complete the questionnaire adequate?** | 4.231 | 0.942 |
| **2. Is the language used easy to read and understand?** | 4.731 | 0.660 |
| **3. Is it complete, including what it is necessary to find out about my habits?** | 4.500 | 0.672 |
| **4. Is it pleasant to fill in?** | 4.442 | 0.958 |
| **5. Is it easy to receive and send?** | 4.808 | 0.561 |
| **6. Are the instructions clear?** | 4.846 | 0.500 |
| **7. Please, tell us if you have had any difficulties in filling in or understanding any questions (Please, describe the question number and the difficulty encountered)** |  |  |
| **8. Please, if you wish you may suggest any more questions or issues that you think should be included in the questionnaire**  **9. Have you detected any error or item that is difficult to respond? #**  **10. In your view, what aspects, topics or items need to be included that are either not present in the questionnaire or not adequately included? #**  **11. Please provide any suggestions for improvement that you would like us to consider. #** |  |  |

^NOTE^: **#** Questions addressed only to the nominal group

**Table S3**: Participant’s socio-demographic characteristics (N = 571).

|  |  | **N** | **%** |
| --- | --- | --- | --- |
| **AGE** (M 30.3, SD 7.5, Range 18-45) | | | |
| **Sex** | Male | 104 | 18.2 |
|  | Female | 467 | 81.8 |
| **Education** | No studies | 0 | 0 |
|  | Primary school | 0 | 0 |
|  | Vocational training | 92 | 16.1 |
|  | Secondary school | 99 | 17.3 |
|  | University degree | 204 | 35.7 |
|  | Master degree | 147 | 25.7 |
|  | PhD | 16 | 2.8 |
| **Salary** | Do not know/No answer | 53 | 9.3 |
|  | < 550 € | 16 | 2.8 |
|  | 550 – 1050 € | 35 | 6.1 |
|  | 1.050 - 1.550 € | 98 | 17.2 |
|  | 1.550 - 2.200 € | 127 | 22.2 |
|  | 2.200 - 3.600 € | 140 | 24.5 |
|  | 3.600 - 5.000 € | 72 | 12.6 |
|  | > 5.000 € | 30 | 5.3 |
| **Body Mass Index:** M 23.76 (SD 4.75; Range 15.4-55.1) | | | |

**Table S4: Item descriptive statistics (n = 571)**

| **ITEM** | **QUESTION** | **LOADING** |  | **I-CVI** |
| --- | --- | --- | --- | --- |
| Q1 | How many meals do you eat a day? | 0.542** |  | 1 |
| Q2 | How often do you eat whole FRUIT (excluding juices, smoothies or puree)? | 0.490** |  | 1 |
| Q3 | How often do you eat VEGETABLES and GREENS? | 0.318** |  | 1 |
| Q4 | How many times a week do you consume dairy products? | 0.309** |  | 0.86 |
| Q5 | How many times a week do you eat CEREALS? | 0.674** |  | 1 |
| Q6 | How many times a week do you eat LEGUME? | 0.345** |  | 1 |
| Q7 | Do you drink SUGARED REFRESHMENTS? | 0.601** |  | 0.86 |
| Q8 | How often do you eat "fast food"? | 0.709** |  | 1 |
| Q9 | How often do you eat fried foods? | 0.604** |  | 0.86 |
| Q10 | How often do you consume ULTRAPROCESSED products? | 0.679** |  | 0.86 |
| Q11 | How many times a week do you eat WHITE FISH? | 0.771** |  | 0.86 |
| Q12 | How many times a week do you eat BLUE FISH including canned? | 0.616** |  | 0.86 |
| Q13 | How many times a week do you eat WHITE MEAT? | 0.283** |  | 0.86 |
| Q14 | How many times a week do you eat RED MEAT? | 0.187* |  | 0.86 |
| Q15 | How often do you have thoughts related to "feeling fat", or fear of gaining weight? | 0.928** |  | 0.86 |
| Q16 | How often do you feel that you have no control over the amount of food you eat or feel guilty or ashamed after eating? | 0.853** |  | 0.86 |
| Q17 | How often are you concerned about your body shape? | 0.892** |  | 0.86 |
| Q18 | How many hours a day do you usually sleep? | 0.518** |  | 1 |
| Q19 | Assess the quality of your sleep | 0.863** |  | 0.86 |
| Q20 | How often do you wake up rested? | 0.553** |  | 0.86 |
| Q21 | How many times a week do you usually go out at night? | 0.613** |  | 0.86 |
| Q22 | How often do you consume alcoholic drinks? | 0.743** |  | 1 |
| Q23 | How often do you have 6 or more alcoholic drinks on one drinking occasion? | 0.901** |  | 0.86 |

*Note: ** p < 0.001; *p < 0.01*

**Table S5**. Scores, reliability and correlations of NutSo-HH factors and IASE index (n = 571)

|  | **Mean (Range; SD)** | **ꭥ** | **F1** | **F2** | **F3** | **F4** | **F5** | **F6** | **NUTRI** | **HH** |
| --- | --- | --- | --- | --- | --- | --- | --- | --- | --- | --- |
| F1 | 2.96 (1.5-3.8; 0.43) | 0.60 | - |  |  |  |  |  |  |  |
| F2 | 2.91 (1.2-4.0; 0.49) | 0.77 | 0.319** | - |  |  |  |  |  |  |
| F3 | 2.24 (1-3.3; 0.52) | 0.57 | 0.056 | 0.149** | - |  |  |  |  |  |
| F4 | 2.61 (1-4; 1.00) | 0.92 | 0,129** | 0.207** | 0,070 | - |  |  |  |  |
| F5 | 2.77 (1-4; 0.53) | 0.70 | 0.127** | 0.093* | -0.033 | 0.294** | - |  |  |  |
| F6 | 3.65 (1.7-4; 0.41) | 0.79 | 0.032 | 0.179** | 0.074 | 0.053 | -0110** | - |  |  |
| Nutri | 2.69 (1.3-3.7; 0.39) | 0.70 | 0.290** | 0.897** | 0.571** | 0.203** | 0.062 | 0.181** | - |  |
| HH | 2.69 (1-3.8; 0.63) | 0.78 | 0.156** | 0.203** | 0.041 | 0.916** | 0.652** | -0.004 | 0.187** | - |
| IASE | 54.12 (21.2-72; 9.6) |  | 0.728** | 0.371** | 0.310** | 0.162^**^ | 0.109** | 0.020 | 0.446** | 0.174** |

NOTE: F1 = Mediterranean foods; F2 = Healthy and unhealthy foods; F3 = Meat and dairy products; F4= Eating disorders; F5 = Alcohol consumption; F6 = Rest habits; NUTRI = Nutritional habits; HH = Health Habits; ꭥ = McDonald's ꭥ; ** p < 0.01 (two tailed); * p < 0.05 (two tailed).
