# Supplementary material for: Development and psychometric testing of the nutritional and social health habits scale (NutSo-HH): A methodological review of existing tools
Source: MethodsX. 2024 May 22;12:102768. doi: 10.1016/j.mex.2024.102768 (PMC11177200; doi:10.1016/j.mex.2024.102768)
Supplement: Supplementary file 2 [file mmc2.docx]

Supplementary File 2: NutSo-HH questionnaire

**I affirm that I understand the purpose of this study, agree to voluntarily answer the questionnaire and consent to the use of the data in this research.**

YES NO

**SOCIO-DEMOGRAPHIC DATA**

**Sex**

Female Male

**Age**

(Whole number)

**Place of birth (province)**

(fold-out with the provinces of Spain)

**Place of residence (province)**

(fold-out with the provinces of Spain)

**How big is the municipality where you live?**

Under 2,000 inhabitants

Between 2,000 and 10,000 inhabitants

Over 10,000 inhabitants

**Current weight (kg)**

(Number)

**How tall are you? (cm)**

(Number)

**What is the highest level of education you have completed or passed?**

No education

Primary school

Secundary school

Professional training

Degree

Master

PhD

**In the last year have you been in paid employment for at least 6 months?**

YES NO

**What is the net monthly income level of your household?**

Less than 550 euros

From 550 to 1050 euros

From 1050 to less than 1550 euros

From 1550 to less than 2200 euros

From 2200 to less than 3600 euros

From 3600 to less than 5000 euros

More than 5000 euros

Don't know / No answer

**How do you consider your general health?**

0 1 2 3 4 5

Very bad Very good

**Where do you live?**

Family home

With relatives outside the home

Only in a flat

Shared flat with roommates

Rented room

Residence

Other

**Do you suffer from any illness that requires a special diet?**

No

Yes, I am diabetic

Yes, I have coeliac disease

Yes, I am lactose intolerant

Yes, I am fructose intolerant

Yes, I have nut allergies

Yes, I am allergic to shellfish

Yes, I am allergic to eggs

Yes, I have another type of allergy or intolerance

Other (other diseases requiring a special diet)

**Do you have a diagnosed eating disorder?**

No

Yes, anorexia nervosa

Yes, bulimia nervosa

Yes, binge eating disorder

Yes, orthorexia (the obsession with healthy eating)

Yes, vigorexia (obsession with physical exercise)

Yes, several at once

Yes, other

**Do you follow any special diet on a regular basis (excluding diets followed on an ad hoc basis to lose weight)?**

No

Yes, vegan

YES, ovo-lacto-vegetarian

Yes, Ovo-vegetarian

Yes, Lacto-vegetarian

Yes, Crudivore

Yes, Frugivore Diet

Yes, Ketone Diet

Yes, Paleo Diet

Yes, Weight Watchers Diet

Yes, Flexitarian Diet

Yes, Intermittent Fasting Diet

Yes, Other

**NUTRITIONAL AND SOCIAL HABITS**

**1. How many meals do you eat per day?**

Lunch and dinner (2 per day)

Breakfast and lunch (2 per day)

Breakfast or mid-morning, lunch and dinner (3 per day)

Breakfast and/or mid-morning, lunch, afternoon snack, dinner (4-5 per day)

I eat more than 5 meals a day

I do long periods of fasting

**2. How often do you eat whole FRUIT (excluding juices, smoothies or puree)?**

Never or rarely

1 piece/ration per week

Between 2 and 4 pieces/rations per week

5 or more pieces/rations per week

Every day

**3. How often do you eat VEGETABLES and VEGETABLES?**

Never or rarely

1 piece/ration per week

Between 2 and 4 pieces/rations per week

5 or more pieces/rations per week

Every day

**4. How many times a week do you consume Dairy products (Milk, Yoghurt, Cheese etc.)?**

Never or rarely

1-2 times a week

3 or more times a week

Every day

**5. How many times a week do you eat CEREALS (rice, maize, wheat, rye, oats etc.)?**

Never or rarely

1-2 times a week

3 or more times a week

Every day

**6. How many times a week do you eat pulses (chickpeas, lentils, beans, etc.)?**

Never or rarely

1-2 times a week

3 or more times a week

Every day

**7. Do you drink sweetened REFRESHMENTS (including light and regular soft drinks, and brick juices)?**

Never

Very seldom (2 times a month maximum)

One glass per week

2 or more glasses per week

2 glasses or less every day

3 to 5 glasses every day

More than 5 glasses every day

**8. How often do you eat "fast food" (pizza, hamburgers, hot dogs, sandwiches) or ready meals?**

Never

Very seldom (2 times a month maximum)

Once a week

Several times a week

Every day

**9. How often do you eat "FRIED" (croquettes, potatoes, fried fish, squid rings, onion rings etc.)?**

Never

Very seldom (2 times a month maximum)

Once a week

Several times a week

Every day

**10. How often do you consume ultra-processed food (jelly beans, chocolate bars, various snacks, etc.)?**

Never

Very seldom (2 times a month maximum)

Once a week

Several times a week

Every day

**11. How many times a week do you eat WHITE FISH (Blue whiting, Sea bream, John Dory, Sole, Hake, Grouper, Pink, Anglerfish etc.)?**

Never or rarely

1-2 times a week

3 or more times a week

Every day

**12. How many times a week do you eat BLUE FISH including canned fish (sardine, mullet, tuna, salmon, trout, bonito, swordfish, turbot, mackerel, anchovy, permit, eel, herring, carp, horse mackerel, elver etc.)?**

Never or rarely

1-2 times a week

3 or more times a week

Every day

**13. How many times a week do you eat WHITE MEAT (chicken, rabbit, pork loin, turkey, lamb)?**

Never or rarely

1-2 times a week

3 or more times a week

Every day

**14. How many times a week do you consume RED MEAT (Beef, Pork, Ox, Horse or Foal, Poultry, Duck and Goose, Goat meat, Lamb)?**

Never or rarely

1-2 times a week

3 or more times a week

Every day

**15. How often do you have thoughts related to "feeling fat", or fear of gaining weight?**

Never

Rarely

Occasionally

Frequently

Very frequently

Always

**16. How often do you feel that you have no control over the amount of food you eat or feel guilty or ashamed after eating?**

Never

Rarely

Occasionally

Frequently

Very frequently

Always

**17. How often are you concerned about your body shape?**

Never

Rarely

Occasionally

Frequently

Very frequently

Always

**18. How many hours a day do you usually sleep? ***

Less than 6 hours

Between 6 and 7 hours

Between 7 and 7.5 hours

Between 7.5 and 8 hours

More than 8 hours

**19. Assess your sleep quality ***

0 1 2 3 4 5

Very bad Very good

**20. How often do you wake up rested? ***

Never

Rarely

Occasionally

Frequently

Very frequently

Always

**21. How many times a week do you usually go out at night? ***

Never

Rarely

Saturday

Friday and Saturday

More than 3 times a week

Every day

**22. How often do you drink alcoholic beverages? ***

Never

1 time per month

2 to 4 times a month

2 to 3 times a week

4 or 5 times a week

Daily

**22. How often do you have 6 or more alcoholic drinks on one drinking occasion? ***

Never

Less than once a month

Monthly

Weekly

Daily or almost daily
